# Carbon dioxide levels in initial nests of the leaf-cutting ant *Atta sexdens* (Hymenoptera: Formicidae)

**DOI:** 10.1038/s41598-021-00099-8

**Published:** 2021-10-18

**Authors:** Kátia K. A. Sousa, Roberto S. Camargo, Nadia Caldato, Adriano P. Farias, Carlos A. O. Matos, José C. Zanuncio, Isabel C. L. Santos, Luiz C. Forti

**Affiliations:** 1 Departamento de Proteção Vegetal, Universidade Estadual Paulista (UNESP), Faculdade de Ciências Agronômicas, Botucatu, 18603-970 Brazil; 2 Campus Experimental de Itapeva, Universidade Estadual Paulista, Itapeva, 18409-010 Brazil; 3 Departamento de Entomologia/BIOAGRO, Universidade Federal de Viçosa, Viçosa, 36570-900 Brazil; 4 Laboratório de Fitossanidade (FitLab), Instituto Federal de Mato Grosso, Cáceres, 78201-380 Brazil

**Keywords:** Ecology, Evolution, Ecology

## Abstract

Claustral foundation of nests by *Atta sexdens* Forel (Hymenoptera: Formicidae) involves great effort by its queens, solely responsible for the cultivation of the fungus and care for her offspring at this stage. The minimum workers, after 4 months, open access to the external environment to foraging plants to cultivate the symbiotic fungus, which decomposes the plant fragments and produces gongilidea nodules as food for the individuals in the colony. Colony gas exchange and decomposition of organic matter in underground ant nests generate carbon dioxide (CO_2_) emitted into the atmosphere. We described the carbon dioxide concentration in colonies in the field. The objective was to evaluate the carbon dioxide concentration in initial *A. sexdens* colonies, in the field, and their development. The CO_2_ level was also measured in 4-month-old colonies in the field, using an open respirometric system fitted with an atmospheric air inlet. The CO_2_ level of the respirometric container was read by introducing a tube into the nest inlet hole and the air sucked by a peristaltic pump into the CO_2_ meter box. The CO_2_ concentration in the initial colony was also measured after 4 months of age, when the offspring production (number of eggs, larvae, pupae and adult workers) stabilized. Ten perforations (15 cm deep) was carried out in the adjacent soil, without a nest of ants nearby, to determine the concentration of CO_2_. The composition of the nests in the field was evaluated after excavating them using a gardening shovel and they were stored in 250 ml pots with 1 cm of moistened plaster at the bottom. The CO_2_ concentration was higher in field nest than in adjacent soil. The concentration of carbon dioxide in *A. sexdens* nests in the field is higher than in those in the soil, due to the production of CO_2_ by the fungus garden and colony.

## Introduction

Leaf-cutting ants are the main herbivores in the Americas, from the southern United States (USA) to central Argentina, with underground nests sheltering colonies with large numbers of individuals that forage fresh vegetation^1^. These nests, annually, release reproductive castes that will originate new nests^2,3^. The foundation of claustral *Atta sexdens* Forel (Hymenoptera: Formicidae) nests involves a great effort by the queen as the only responsible to cultivate the fungus and caring for her offspring in this phase^3,4^.

About 3 to 4 months, after the claustral phase, average workers from initial nests clear the channel closed by the founding queen to the soil surface^5^. Access to the external environment allows ants to forage plant material to cultivate the symbiotic fungus *Leucoagaricus gongylophorus* (A. Møller) Heim)^6^. This fungus breaks down plant fragments and produces hyphal nodules (gongilids) that will serve as food for the individuals of the colony^7^.

Nesting in deep soil layers facilitates the increase in the concentration of carbon dioxide (CO_2_)^8^. The large volumes of this gas, produced by the ant respiration and the decomposition of organic matter, contribute to its emission from underground nests into the atmosphere^9,10^. CO_2_ concentrations in field nests were 0.2% in *Pogonomyrmex badius* (Latreille)^11^, 1.5 to 4.5% in *A. capiguara* (Gonçaves) and *A. laevigata* (Smith), respectively^8^ and 5.7% in *A. vollenweideri* (Forel)^12^, exceeding atmospheric levels of this gas which is 0.04%. The high levels of carbon dioxide in the *A. vollenweideri* nest, native to clayey soils, are a problem. The porosity and air permeability of these soils are low^13^, hindering the release of CO_2_ from the chambers and the supply of oxygen in the nest by diffusion with the surrounding soil. The wind-induced ventilation mechanism, according to the Bernoulli^14^ principle, facilitates gas exchange in *A. vollenweideri* nests, similar to the ventilation of the *Cynomys ludovicianus*^15^ rodent burrows. These nests have up to 200 openings used as exits or entrances by the ants^16^ and the entrance and exit of air through them depend on their location in the soil mound. Surface wind drives air out of the central tunnels, followed by an inflow of air at the periphery. Ants build towers on the top of central openings of the nests for ventilation raising the tunnel opening and exposing them to higher surface wind speeds^14^. This gas exchange in adult ant nests is best studied, but this is poorly understood in early ones.

An organism's aerobic respiration releases CO_2_, but its rate of production expelled in early leaf-cutting ant colonies is unknown. The objective was to study the carbon dioxide concentrations in initial colonies of *A. sexdens*, in the field and their development.

## Results

**Figure 1:**
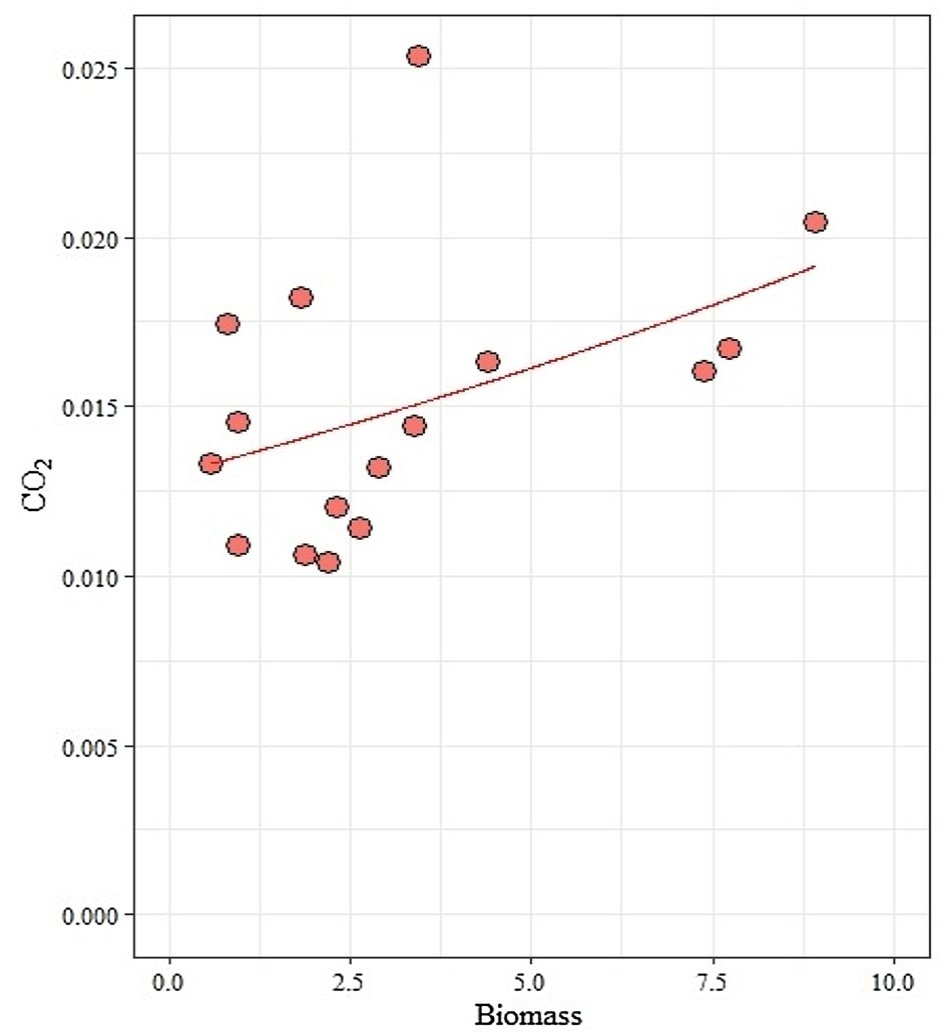
Relation between CO_2_ production and biomass of early colonies of *Atta sexdens* (Hymenoptera: Formicidae) in soil.

CO_2_ emission by *A. sexdens* nests (median = 1.40%) was higher than adjacent soil (Wilcoxon rank test pval < 0.05). The CO_2_ emission was related to fungus biomass higher (Estimated Coefficient = 0.04426, p < 0.05) (Fig. 1).

The CO_2_ model = f(biomass) (Coefficient = 0.04426, p < 0.05) of CO_2_ emission in field colonies (Table 1). Fungus biomass, queen mass, number of eggs and mean workers showed values of 4.70 ± 5.05, 206.73 ± 23.45 mg, 144.33 ± 66.31, 119.61 ± 79.34, and 25.67 ± 9.91. See more details in the Supplementary Information.

**Table 1:** Summary of estimates of model coefficients CO_2_ = f(biomass)—field.

Field				
Model with logit link)	Estimate	Std. error	z	value Pr( >|z|)
Coefficients (mean: intercept model with logit link): biomass	− 4.33144	0.09274	− 46.705	< 2e−16
	0.04426	0.02082	2.126	0.0335
**Phi coefficients (precision model with identity link)**				
(Phi)	1353.5	480.3	2.818	0.00483

Laboratory: Log-likelihood: 155.3 on 3 Df, Pseudo R^2^: 0.01306; Field: Log-likelihood: 68.98 on 3 Df, Pseudo R^2^: 0.2073.

## Discussion

The greater CO_2_ emission by *A. sexdens* nests in the field than in those in the adjacent soil. Early nests of *A. sexdens* are found at a depth of around 15 cm, where soils are one of the largest global reserves of carbon. In addition, soil fauna alters the structure of soil processes by stimulating or inhibiting the CO_2_ flow^17,18^. This may explain the greater emission of this gas by initial field nests is higher than soil matrix. However, the deepening of early *Atta* nests with greater numbers of chambers and tunnels increases soil manipulation and, consequently, the release of gases with the amount of CO_2_ from adult ant nests and surrounding soils being 15% to 60% higher than those more distant^18^.

Differences in the CO_2_ model when biomass and CO_2_ emission in field colonies were used are due to the greater emission of this gas by the soil microbiota. Therefore, field nestsare exposed to high concentrations of carbon dioxide^10,18,19^. Climatic parameters such as humidity, temperature and CO_2_ concentration directly impact the development of the ant nest^20^. CO_2_ levels in nests of *Acromyrmex lundi* Guering (Hymenoptera: Formicidae) were between 1 and 3% and their workers avoided high levels of this gas^21^. CO_2_ values in giant nests such as *A. vollenweideri* did not exceed 28,000 ppm due to small towers to facilitate nest ventilation and carbon dioxide removal when their levels are above 5%^22^. Ants elicit specific reactions to CO_2_, through sensillum ampullaceum, embedded below the cuticle of the antenomer and harboring cells that receive this gas^23,24^.

The numbers of eggs, larvae, pupae and small and medium workers in *A. sexdens* colonies in field is similar to found in literature for *Atta sexdens*^25^. The factors such as temperature, humidity and plants could affect the development of fields colonies^32^. In the field is dement the higher energy used to excavate them and form the first chamber^25–28^.

The higher concentration of carbon dioxide in *A. sexdens* nests in the field than in the adjacent soil is due to the fact that the initial nest chamber in natural conditions produce more CO_2_^18^ by fungus garden and colony. In addition, Biogenic sources of CO_2_ can be due to decomposition of plant matter, root exudation or addition of plant residues, microbial decomposition of soil organic matter, root respiration and rhizomicrobial respiration (or fine root decomposition)^29^.

## Methods

### CO_2_ levels of field colonies

The study was carried out at Fazenda Santana near UNESP Experimental Farm Lageado, municipality of Botucatu, state of São Paulo, Brazil (22°50′46″S and 48°26′02″W). Initial nests of *A. sexdens* were marked and the CO_2_ concentration measured in them.

**Figure 2:**
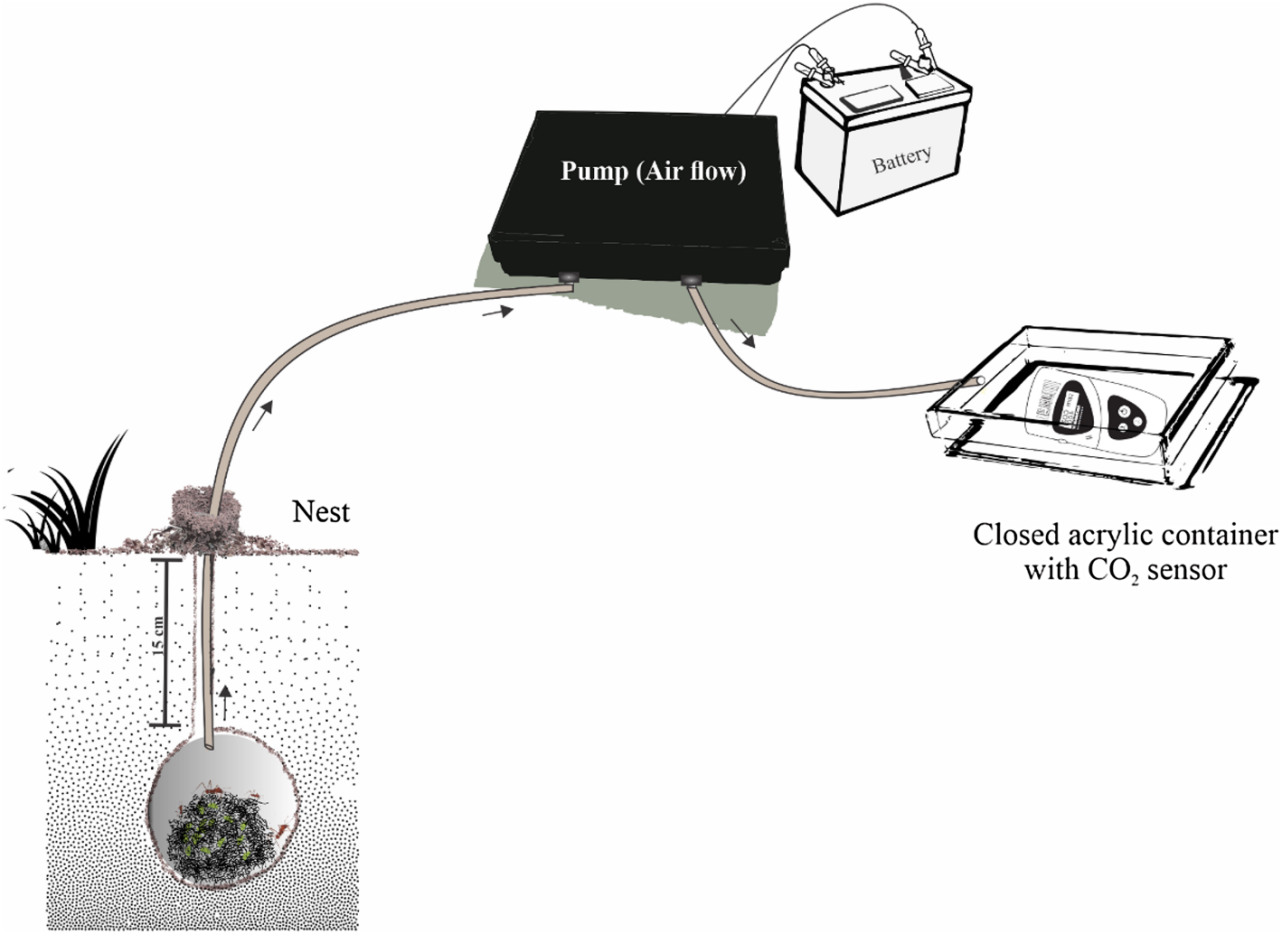
Scheme of the closed respirometric system used in the experiment to measure CO_2_ in nests of *Atta sexdens* (Hymenoptera: Formicidae) in field. Design created by Kátia K. A. Sousa by CorelDRAW 2019 (https://www.coreldraw.com/br/?link=wm).

An open respirometric system was built and adapted (Fig. 2) with atmospheric air inlet and the CO_2_ level of the respirometric container (Bacharach) measured with a fixed probe (http://www.bacharach-32.inc.com). This measurement was carried out by introducing a tube into the nest inlet hole and the air sucked by a peristaltic pump into the CO_2_^30^ meter box.

The nest holes were closed for 24 h after CO_2_ measurement and opened after this time when this gas were measurements again.

In addition, ten perforations (15 cm deep) was carried out in the adjacent soil, without a nest of ants nearby, to determine the concentration of CO_2_.

### Composition of the initial colony

The composition of the initial colony was evaluated by counting the number of eggs, larvae, pupae and adult workers in 4-month-old *A. sexdens* nests in the field. This composition was obtained by excavating the nests using gardening shovel and storing them in 250 ml pots with 1 cm of moistened plaster at the bottom. The offspring of each colony were counted in the laboratory under a stereomicroscope.

### Statistical analysis

Statistical analyzes of proportions of CO_2_ production were limited between 0 and 1 with variability, commonly, according to the mean of the response, not meeting the assumptions of normal distribution of residues and homogeneous variance of standard techniques of statistical analysis. Beta regression is an easier and more flexible interpretation method than transformations (sine arc(root(Y/100)), etc.) to model proportions originating from continuous measures limited to the open interval (0,1) whose most important aspects are identified by those familiar with generalized linear models (GLMs)^31–33^. The mean-precision parameterization, with µ (for the expected value) and ϕ (as a measure of 'precision', or the inverse of dispersion), is most commonly used in the context of beta regression) Maximum likelihood estimation method of β and ϕ is used to best fit the data to the model. The estimated coefficients of the model are related to the linear predictor in the transformed scale:

**Table:** 

(Intercept)	X	(phi)
− 4.33144	0.04426	1353.5

The estimated coefficients on the scale of the original observations must be transformed using the inverse of the link function so that the nonlinear relationship on the scale of the original observations is restored. For example, the predicted expected value when X = 1 is:

$$g\left( {E\left[ {Y\left| {X = 1} \right.} \right]} \right) = - 4.33144 + 0.04426*1 = - 4.28718$$

$$E\left[ {Y\left| {X = 1} \right.} \right] = \frac{{e^{ - 4.28718} }}{{1 + e^{ - 4.28718} }} = 0.0135573$$

The hypothesis for medians was tested with the Wilcoxon Rank Sum Test for variables without normal distribution and the means with the homoscedastic t Test for variables with normal distribution. The significance level adopted in this and all other analyzes was 5% (α = 0.05). The analysis of standardized residuals in a contingency table was performed after the Χ^2^ independence test was performed.

## Supplementary Information

Supplementary Information.
