# Supplementary material for: Carbon dioxide levels in initial nests of the leaf-cutting ant Atta sexdens (Hymenoptera: Formicidae)
Source: Sci Rep. 2021 Oct 18;11:20562. doi: 10.1038/s41598-021-00099-8 (PMC8523712; doi:10.1038/s41598-021-00099-8)
Supplement: Supplementary file 1 — Supplementary Information. [file 41598_2021_99_MOESM1_ESM.doc]

**Supplementary Material**

**Carbon dioxide levels in initial nests of the leaf-cutting ant *Atta sexdens* (Hymenoptera: Formicidae)**

**Scientific background**

An organism's aerobic respiration releases CO_2_, but its rate of production expelled in early leaf-cutting ant colonies is unknown. Could CO_2_ levels in early field nests differ from age-matched laboratory colonies? The objective was to study the carbon dioxide concentrations in initial colonies of *A. sexdens*, in the field and laboratory, and their development.

**Results**

CO_2_ emission by *A. sexdens* nests was lower (coef. Phi=-3.70902, p<0.05) (Table 1) and fungus biomass higher (Fig. 1) in laboratory colonies than in field ones with, respectively, 0.04 ± 0.01% and 1.50 ± 0.38%.

**Table 1.** Summary of estimates of model coefficients CO_2_= f(environment) varying with predictors

| Coefficients (mean model with logit link): |  | Estimate | Std. Error | z | value Pr(>|z|) |
| --- | --- | --- | --- | --- | --- |
|  | Intercept | -4.18454 | 0.05916 | -70.74 | <2e-16 |
|  | Laboratory | -3.70902 | 0.08703 | -42.62 | <2e-16 |
| Phi coefficients (precision model with log link): | Intercept | 7.0359 | 0.3445 | 20.423 | < 2e-16 |
|  | Laboratory | 3.3657 | 0.4692 | 7.174 | 7.30E-13 |

Log-likelihood: 227 on 4 Df, Pseudo R-squared: 0.9788


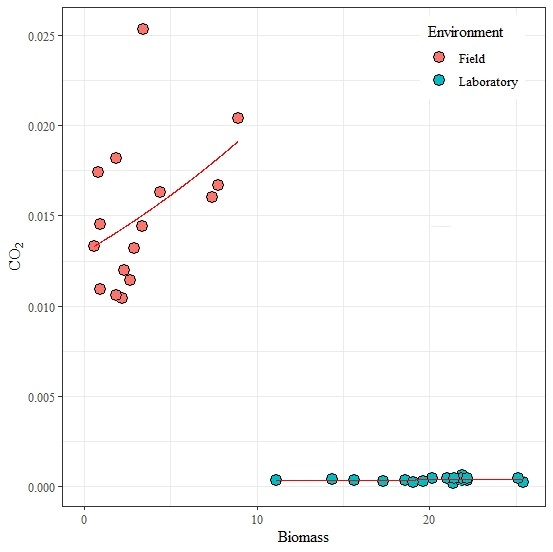


**Figure 1.** Correlation between CO_2_ production and biomass of early colonies of *Atta sexdens* (Hymenoptera: Formicidae) in laboratory and field.

The model of CO_2_= f(biomass) (Coefficient= 0.00956, p=0.611) was similar for fungus biomass and CO_2_ emission in *A. sexdens* colonies in the laboratory (Fig. 1, Table 2). The biomass of the laboratory colonies was 20.18 ± 3.44 (g) and the CO_2_ emission 0.037 ± 0.010%.

**Table 2.** Summary of estimates of model coefficients CO_2_= f(biomass) – laboratory and field

| Laboratory | | | | | | | | | | | |
| --- | --- | --- | --- | --- | --- | --- | --- | --- | --- | --- | --- |
|  | | Estimate | | | Std. Error | | z | | value Pr(>|z|) | | |
| Coefficients (mean Intercept | | -8.08701 | | | 0.38696 | | -20.899 | | <2e-16 | | |
| model with logit link): biomass | | 0.00956 | | | 0.01882 | | 0.508 | | 0.611 | | |
| Phi coefficients (precision model with identity link): | | | | | | | | | | | |
| (phi) | 33358 | | | 10623 | | | | 3.14 | | | 0.00169 |
| Field | | | | | | | | | | | |
| model with logit link): | | Estimate | Std. Error | | | z | | | | value Pr(>|z|) | |
| Coefficients (mean Intercept | | -4.33144 | 0.09274 | | | -46.705 | | | | <2e-16 | |
| model with logit link): biomass | | 0.04426 | 0.02082 | | | 2.126 | | | | 0.0335 | |
| Phi coefficients (precision model with identity link): | | | | | | | | | | | |
| (phi) | 1353.5 | | | 480.3 | | | | 2.818 | | | 0.00483 |

Laboratory: Log-likelihood: 155.3 on 3 Df, Pseudo R-squared: 0.01306; Field: Log-likelihood: 68.98 on 3 Df, Pseudo R-squared: 0.2073

The CO_2_ model= f(biomass) (Coefficient= 0.04426, p<0.05) differed when using the fungus biomass and CO_2_ emission in field colonies (Table 3), with values ​​of 4.70 ± 5.05 (g) and of 1.498 ± 0.359%, respectively.

**Table 3.** Hypothesis tests for similarity between medians (Wilcoxon Rank Sum Test) and means (t Test) of fungus biomass (Bio.) (g), queen mass (QM) (mg) and egg numbers (Egg), larvae (La.), pupae (Pu.), small (SW) and medium (MW) workers and total workers (TW) in nests of *Atta sexdens* (Hymenoptera: Formicidae) in laboratory and field

|  | Laboratory | Field | Test | Value P |
| --- | --- | --- | --- | --- |
| Bio. | 21.40 | 2.65 | W= 332 | 0.000 |
| QM | 270.09 | 207.45 | t= 6.5374 df =35 | 0.000 |
| Egg | 271.00 | 133.00 | W=282 | 0.001 |
| La. | 171.50 | 106.00 | W=236 | 0.046 |
| Pu. | 81.00 | 78.00 | W=169 | 0.988 |
| SW | 127.5 | 98.00 | W=219,5 | 0.135 |
| MW | 36.50 | 24.00 | W=277 | 0.001 |
| TW | 164.5 | 121.00 | W=250 | 0.015 |

* Mean

The numbers of eggs, larvae, pupae and small and medium workers were correlated in the laboratory and field colonies (Pearson's Chi-squared test X-squared= 28,158, df= 4, p-value= 1.159e-05). Fungus biomass, queen mass, number of eggs and mean workers differed between colonies in laboratory and field (Table 4, Fig. 2) with values ​​of 20.18 ± 3.44 and 4.70 ± 5.05, 279.09 ± 32.71 and 206.73 ± 23.45 mg, 293.10 ± 138.03 and 144.33 ± 66.31, 169.80 ± 65.16 and 119.61 ± 79.34, and 43.10 ± 22.90 and 25.67 ± 9.91, respectively.

**Table 4**. Pearson linear correlation coefficient between fungus biomass (Bio.) (g), queen mass (QM) (mg) and egg numbers (Egg), larvae (La.), pupae (Pu.), small (SW) and medium (MW) workers and total workers (TW) in nests of *Atta sexdens* (Hymenoptera: Formicidae) in the field and 4laboratory.

| Field colonies |  | Egg | La. | Pu. | QM | SW | MW |
| --- | --- | --- | --- | --- | --- | --- | --- |
|  | Egg |  |  |  |  |  |  |
|  | La. | **0.77** |  |  |  |  |  |
|  | Pu. | **0.86** | **0.8** |  |  |  |  |
|  | QM | 0.27 | 0.25 | 0.34 |  |  |  |
|  | SW | **0.85** | **0.75** | **0.85** | 0.46 |  |  |
|  | MW | 0.61 | 0.65 | 0.68 | 0.35 | **0.82** |  |
|  | Bio | 0.6 | 0.36 | 0.53 | 0.38 | 0.42 | 0.13 |
|  |  |  |  |  |  |  |  |
| Laboratory colonies |  | Egg | La. | Pu. | QM | SW | MW |
|  | Egg |  |  |  |  |  |  |
|  | La. | **0.74** |  |  |  |  |  |
|  | Pu. | 0.19 | 0.41 |  |  |  |  |
|  | QM | 0.08 | -0.2 | -0.18 |  |  |  |
|  | SW | 0.24 | 0.32 | 0.27 | -0.16 |  |  |
|  | MW | **-0.46** | -0.22 | -0.3 | -0.25 | -0.18 |  |
|  | Bio | -0.35 | 0.09 | 0.19 | -0.17 | -0.06 | 0.14 |

Bold values are significatives (α= 0.05)

**
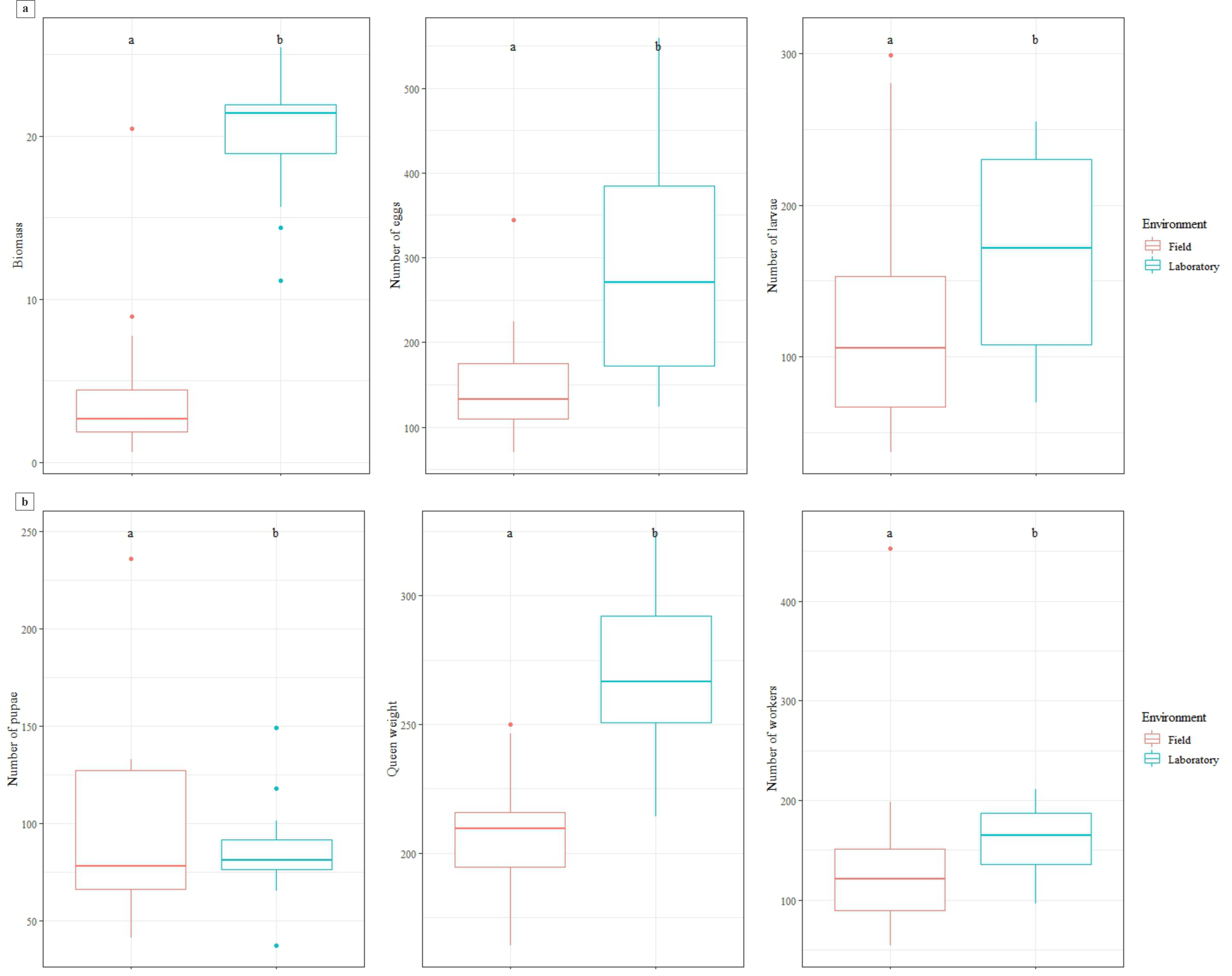
**

**Figure 2.** Composition of early nests of *Atta sexdens* (Hymenoptera: Formicidae) in laboratory (a) and field (b) at four months old.

The numbers of eggs and larvae (r^2^= 0.77), eggs and pupae (r^2^= 0.86), eggs and small workers (r^2^= 0.85), larvae and pupae (r^2^= 0.80), larvae and small workers (r^2^= 0.75) pupae and small workers (r^2^= 0.85) and small workers and medium workers (r^2^= 0.82) were positively correlated in field colonies. The numbers of eggs and larvae were positively correlated (r^2^=0.74) and the numbers of eggs and middle workers negatively correlated (r^2^= -0.46) in laboratory colonies.

The concentration of carbon dioxide in field colonies was higher than that of age-matched laboratory ones.

**Methods**

**Collecting *Atta sexdens* females after the nuptial flight.** Queens of *A. sexdens* were collected at the Experimental Farm Lageado in Botucatu, Brazil in 2018 (22°50'37.3"S 48°25'38.3"W) during this ant nuptial flight on sunny days after heavy rains in October and early November. These queens, which had removed their wings, that is, fertilized, were collected and kept in 250 ml pots with 1 cm of moistened plaster at the bottom. Twenty initial laboratory-maintained colonies and 18 initial colonies collected in the field at four months of age after the nuptial flight were studied. We had all permissions for the collection of  *Atta sexdens* queens specimens.

**CO_2_ levels of laboratory colonies.** The colonies of *A. sexdens*, with four months of age, were enclosed in hermetic chambers for 24 hours, simulating the claustal foundation in a natural environment. CO_2_ emission was measured by a closed respirometric system (Fig. 3) with a CO_2_ meter (Bacharach) with a fixed probe (http://www.bacharach-inc.com) at 25 °C in the laboratory. This environment was fed back by a peristaltic pump, preventing the losses of the modified atmosphere of the *A. sexdens* colony chamber.

**CO_2_ levels of field colonies.** The study was carried out at Fazenda Santana near UNESP Experimental Farm Lageado, municipality of Botucatu, state of São Paulo, Brazil (22°50’46”S and 48°26’02”W). Initial nests of *A. sexdens* were marked and the CO_2_ concentration measured in them.

An open respirometric system was built and adapted (Fig. 3) with atmospheric air inlet and the CO_2_ level of the respirometric container (Bacharach) measured with a fixed probe (http://www.bacharach-32 inc.com). This measurement was carried out by introducing a tube into the nest inlet hole and the air sucked by a peristaltic pump into the CO_2_ meter box.

The nest holes were closed for 24 hours after CO_2_ measurement and opened after this time when this gas were measurements again.


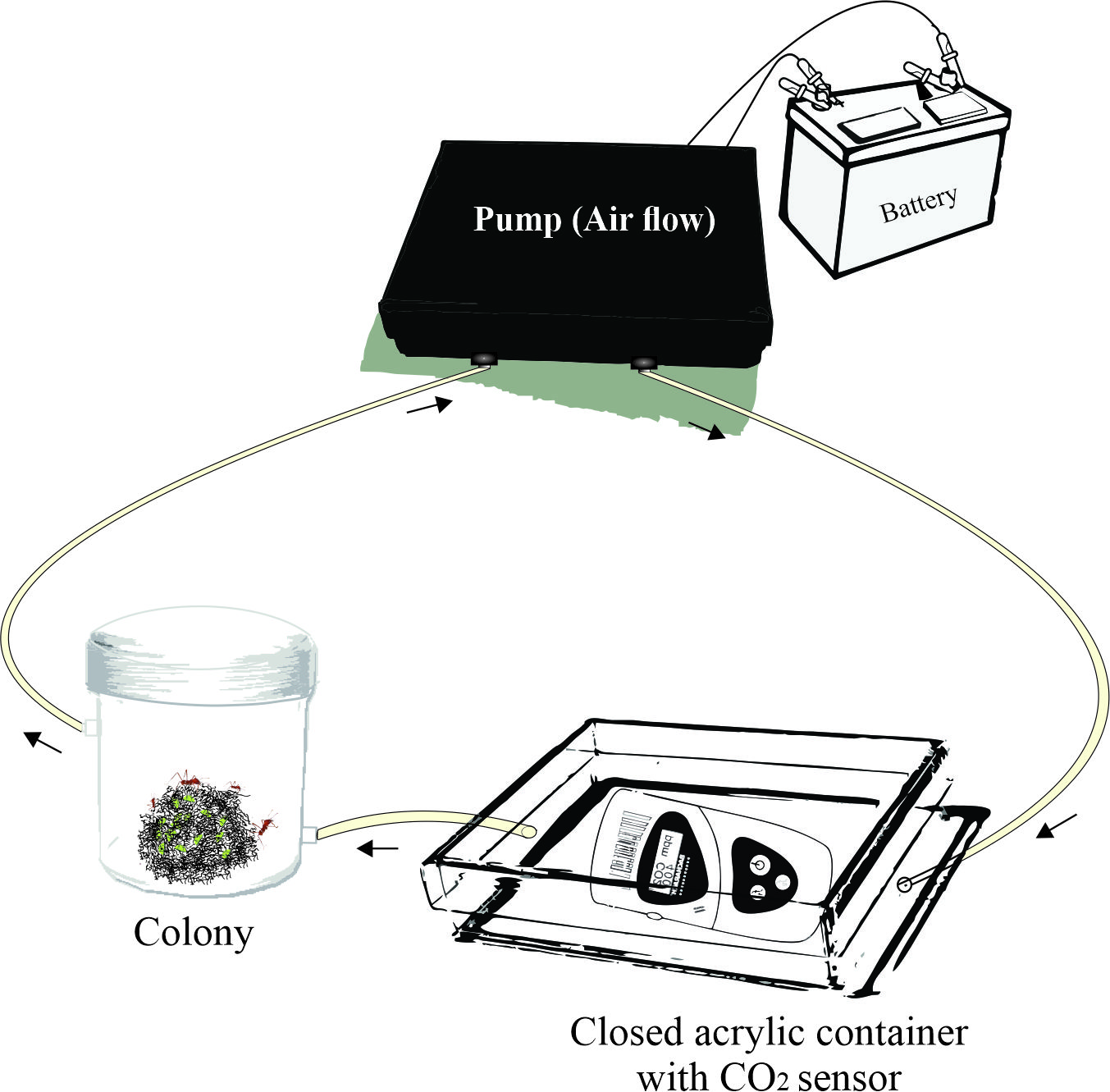
**Figure 3.** Scheme of the closed respirometric system used in the experiment to measure CO_2_ in nests of *Atta sexdens* (Hymenoptera: Formicidae) in laboratory. Design created by Kátia K. A. Sousa by CorelDRAW 2019 (https://www.coreldraw.com/br/?link=wm).

**Composition of the initial colony.** The composition of the initial colony was evaluated by counting the number of eggs, larvae, pupae and adult workers in four-month-old *A. sexdens* nests in the field. This composition was obtained by excavating the nests using gardening shovel and storing them in 250 ml pots with 1 cm of moistened plaster at the bottom. The offspring of each colony were counted in the laboratory under a stereomicroscope.

**Statistical analysis. Statistical analyzes of proportions of CO_2_ production were limited between 0 and 1 with variability, commonly, according to the mean of the response, not meeting the assumptions of normal distribution of residues and homogeneous variance of standard techniques of statistical analysis. Beta regression is an easier and more flexible interpretation method than transformations (sine arc(root(Y/100)), etc.) to model proportions originating from continuous measures limited to the open interval (0,1) whose most important aspects are identified by those familiar with generalized linear models (GLMs). The mean-precision parameterization, with µ (for the expected value) and ϕ (as a measure of 'precision', or the inverse of dispersion), is most commonly used in the context of beta regression. see Box 1) Maximum likelihood estimation method of β and ϕ is used to best fit the data to the model. The estimated coefficients of the model are related to the linear predictor in the transformed scale:**

| (Intercept) | X | (phi) |
| --- | --- | --- |
| -4.33144 | 0.04426 | 1353.5 |

The estimated coefficients on the scale of the original observations must be transformed using the inverse of the link function so that the nonlinear relationship on the scale of the original observations is restored. For example, the predicted expected value when X= 1 is:


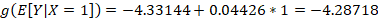


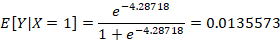


The hypothesis for medians was tested with the Wilcoxon Rank Sum Test for variables without normal distribution and the means with the homoscedastic t Test for variables with normal distribution. The significance level adopted in this and all other analyzes was 5% (α= 0.05). The analysis of standardized residuals in a contingency table was performed after the Χ^2^ independence test was performed. Pearson linear correlation tests were performed with numbers of eggs, larvae, pupae and small and medium workers of *A. sexdens*.
